# Stable Longitudinal Quality of Life in the SERVE Trial Among Adults With Transposition of the Great Arteries and a Systemic Right Ventricle

**DOI:** 10.1016/j.cjcpc.2024.12.001

**Published:** 2024-12-12

**Authors:** Alessandro Castiglione, Markus Schwerzmann, Judith Bouchardy, Ronny Ralf Buechel, Reto Engel, Michael Freese, Harald Gabriel, Matthias Greutmann, Dik Heg, Christian Mueller, Mathias Possner, Francisco Javier Ruperti-Repilado, Tobias Rutz, Jurg Schwitter, Corina Thomet, Daniel Tobler, Matthias Wilhelm, Kerstin Wustmann, Fabienne Schwitz, SERVE trial

**Affiliations:** a Department of Cardiology, Center for Congenital Heart Diseases, Inselspital, Bern University Hospital, University of Bern, Bern, Switzerland; b Division of Cardiology, University of Ottawa Heart Institute, Ottawa, Ontario, Canada; c Department of Cardiology and Cardiac Surgery (CHUV), Centre Hospitalier Universitaire Vaudois, Lausanne, Switzerland; d Division of Cardiology, Hôpitaux Universitaires de Genève (HUG), Genève, Switzerland; e Department of Nuclear Medicine, Cardiac Imaging, University Hospital Zurich, Zurich, Switzerland; f Department of Cardiology, Kantonsspital St. Gallen, St. Gallen, Switzerland; g Department of Cardiology and Cardiovascular Research Institute Basel (CRIB), University Hospital Basel, Basel, Switzerland; h Department of Cardiology, Vienna General Hospital, Medical University of Vienna, Vienna, Austria; i University Heart Center, Department of Cardiology, University of Zurich, Zurich, Switzerland; j Department of Clinical Research, University of Bern, Bern, Switzerland; k Department of Cardiology, University Hospital Basel, University of Basel, Basel, Switzerland; l Cardiac MR Center of the University Hospital Lausanne and CMR Corelab (swissCVIcorelab, CHUV), Lausanne, Switzerland; m University Clinic of Cardiology, Preventive Cardiology and Sports Medicine, Inselspital, University Hospital, Bern, Switzerland; n Department of Congenital Heart Defects and Paediatric Cardiology, German Heart Center Munich, Munich, Germany

## Abstract

**Background:**

Adults with a transposition anatomy and a systemic right ventricle (RV) face long-term complications that may impact their quality of life (QoL). Few data are available regarding the QoL in this patient group and its evolution over time.

**Methods:**

This study was performed in the SERVE trial’s (identifier: NCT03049540) prospective cohort of patients (n = 100) with congenitally corrected transposition of the great arteries (TGA) or dextro-TGA after the atrial switch procedure and a longitudinal follow-up of 3 years. We aimed to describe the longitudinal QoL levels and their predictors. QoL was assessed using the Linear Analog Scale. QoL parameters were collected at baseline, after 12 months, and after 36 months, together with clinical parameters and a questionnaire assessing general self-efficacy (GSE).

**Results:**

The mean QoL on the Linear Analog Scale was 79.1 ± 13.6 at baseline, 75.5 ± 14.8 at 1 year, and 79.2 ± 13.6 at 3-year follow-up (*P* = 0.900). No significant differences in QoL were observed between congenitally corrected TGA or dextro-TGA patients. Cardiopulmonary exercise testing maximum work rate and maximum oxygen uptake, New York Heart Association class, end-diastolic RV volumes, N-terminal pro–B-type natriuretic peptide concentration, and GSE showed significant correlations with QoL levels. Multivariable regression analysis identified GSE value and New York Heart Association class (*r*^2^ = 0.283, *P* < 0.001) as independent predictors of QoL at baseline.

**Conclusions:**

Patients with a systemic RV reported a stable good QoL during 3 years of follow-up. Exercise capacity and self-efficacy were the only independent predictors of QoL.

**Clinical Trial Registration:**

NCT03049540.

## Graphical abstract

**Figure:**
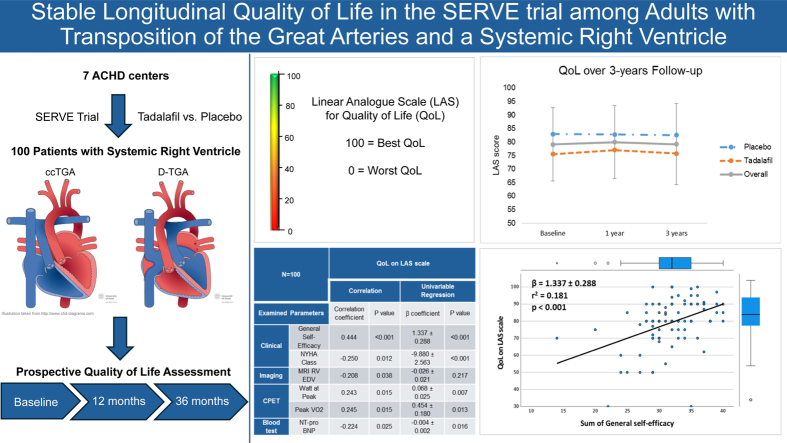

## Résumé

**Contexte:**

Les adultes qui présentent une anatomie de transposition avec un ventricule droit (VD) systémique peuvent connaître à long terme des complications susceptibles de réduire leur qualité de vie (QdV). Il existe par contre peu de données sur la qualité de vie de ces patients et son évolution au fil du temps.

**Méthodologie:**

Cette étude a été menée auprès d’une cohorte prospective de patients (n = 100) de l’essai SERVE (code d’identification: NCT03049540), qui présentaient une transposition congénitalement corrigée des gros vaisseaux (TCCGV) ou une dextrotransposition des gros vaisseaux (dTGV), après une procédure de commutation auriculaire et pendant un suivi longitudinal de 3 ans. Notre objectif était de décrire la QdV longitudinale des patients et les facteurs permettant de la prédire. La QdV a été évaluée à l’aide de l’échelle *Linear Analog Scale* (LAS). Les paramètres liés à la qualité de vie, les paramètres cliniques et les réponses à un questionnaire sur le sentiment d’autoefficacité ont été recueillis au départ, à 12 mois et à 36 mois.

**Résultats:**

La QdV moyenne à l’échelle LAS était de 79,1 ± 13,6 au départ, 75,5 ± 14.8 à 1 an et 79,2 ± 13,6 au suivi de 3 ans (*p* = 0,900). Aucune différence significative n’a été observée sur le plan de la qualité de vie entre les patients présentant une TCCGV et ceux présentant une dTGV. La capacité d’effort maximale et la consommation maximale d’oxygène mesurées lors d’une épreuve d’effort cardiopulmonaire (CPET), la classe selon la New York Heart Association (NYHA), le volume télédiastolique du VD, le taux de propeptide natriurétique de type B N-Terminal (NT-proBNP) et la mesure du sentiment d’autoefficacité présentaient une corrélation significative avec les valeurs de QdV. L'analyse par régression multivariée a permis de constater que la mesure du sentiment d’autoefficacité et la classe selon la NYHA (r^2^ = 0,283, valeur *p* <0,001) étaient des facteurs prédictifs indépendants de la QdV au départ.

**Conclusions:**

Les patients présentant un VD systémique ont signalé une QdV bonne et stable au cours de trois années du suivi. La capacité d’effort et le sentiment d’autoefficacité étaient les deux seuls facteurs prédictifs indépendants de la QdV.

Adults with a systemic right ventricle (RV) have a high risk of developing heart failure and arrhythmias, and may face complex interventions and lifelong medical therapy; all of these can potentially affect their quality of life (QoL).^1^ The concept of QoL has gained prominence in medical research, reflecting the appraisal that the impact of a medical condition extends beyond traditional clinical outcomes.^2^, ^3^, ^4^ As a consequence, studying changes in QoL may reflect a more holistic approach to patient welfare and care.^5^^,^^6^ An important contribution on this topic was the landmark study APPROACH-IS (Assessment of Patterns of Patient-Reported Outcomes in Adults with Congenital Heart disease—International Study).^7^^,^^8^ Data from over 4000 adult congenital heart disease (ACHD) patients in 15 countries over 5 continents were collected to investigate their QoL.^9^ The study provided reliable and up-to-date data on QoL in ACHD patients^7^^,^^10^ but did not differentiate between the specific underlying cardiac defects.

The SERVE trial (Effect of Phosphodiesterase-5 Inhibition With Tadalafil on SystEmic Right VEntricular Size and Function) was a randomized intervention study investigating the effects of phosphodiesterase 5 inhibition on systemic ventricular function in patients with a systemic RV.^11^^,^^12^ As part of the study protocol, QoL and self-efficacy were prospectively assessed during the trial period. The purpose of this predefined substudy was to explore the relationship between QoL, self-efficacy, and clinical parameters in patients with a systemic RV. We aimed to test whether (1) previously identified markers of QoL in ACHD patients also apply to the subgroup of the ACHD population enrolled in the SERVE trial, (2) explore the correlation between QoL and self-efficacy in this patient group, and (3) investigate the longitudinal course of QoL in our patients during the study period.

## Materials and Methods

### The cohort design

The SERVE trial aimed to observe the effect of tadalafil in patients with a systemic RV and a biventricular circulation on right ventricular function. The study included 100 adults with dextro–transposition of the great arteries (d-TGA) after atrial switch operations or with congenitally corrected TGA (ccTGA)^12^ recruited from the participating centers in Switzerland and Austria. Patients with life expectancy below 6 months, patients with severe renal or hepatic dysfunction, and patients with a known intolerance or contraindication to tadalafil were excluded. Other exclusion criteria included incapability of giving informed consent; myocardial infarction, stroke, or open heart surgery within the 3 months before the baseline visit; expected heart transplant within the next 6 months starting from baseline; ongoing pregnancy or breast-feeding; systemic arterial hypotension with noninvasive blood pressures <90/50 mm Hg at the baseline visit; and allergy to iodinated (in patients undergoing coronary multidetector computer tomography) or gadolinium-based (in patients undergoing cardiovascular magnetic resonance [CMR]) contrast agents.^12^ Additional information about design, methods, objectives, and results of the SERVE trials has been published previously in 2 separate papers.^11^^,^^12^ The study is registered at ClinicalTrials.gov (identifier: NCT03049540).

### Objectives and work hypothesis of the present study

First, we aimed to describe the QoL in a well-defined and contemporary subgroup of ACHD patients.

Secondly, we aimed at recognizing clinical predictors of QoL levels in this patient cohort. On the basis of previous publications, we hypothesized that age, patient functional status (expressed as New York Heart Association [NYHA] class), heart function and dimension (systemic ventricular function and size on CMR or computer tomography), cardiorespiratory fitness, cardiac neurohormone plasma levels, and a history of arrhythmias would correlate significantly with QoL levels.^8^^,^^13^, ^14^, ^15^, ^16^ Also, we wanted to investigate whether self-efficacy correlates with QoL in this specific cohort, as self-efficacy was previously found to correlate with QoL in a broader ACHD population.^17^

Lastly, as we observed and reported the QoL change over a prospective 3-year follow-up in the SERVE trial cohort, we tried to identify clinical variables that might correlate with a significant QoL improvement. A lack of a demonstrable effect of the trial treatment with tadalafil on QoL was already presented in the main article discussing the results of the SERVE trial.^11^

### Selection of quality of life and self-efficacy scales

QoL was assessed as in the APPROACH IS.^6^ QoL was explored using 2 tools: the Linear Analog Scale (LAS) for overall QoL and the Satisfaction with Life Scale (SWLS) for global life satisfaction. The validity, temporal stability, and good psychometric qualities of these tools in ACHD have been demonstrated previously.^8^^,^^18^, ^19^, ^20^, ^21^, ^22^, ^23^

The LAS offers a visual analog representation for individuals to rate their well-being, typically on a continuum from “worst imaginable” (score 0) to “best imaginable” (score 100) health state. The SWLS provides a global assessment of life satisfaction through 5 self-reported statements that capture an individual’s cognitive appraisal of their own life.^20^ Here, patients are confronted with a 5-item scale in which the score ranges from 5 (lowest global satisfaction level) to 35 (highest global satisfaction level), whereas a score of 20 represents the neutral point.^20^ For the aim of our study, the LAS was chosen as a primary endpoint to investigate QoL and the SWLS was used as a validation questionnaire.

In addition to QoL, another patient self-reported outcome variable was assessed: self-efficacy. Self-efficacy refers to an individual’s belief in their capacity to execute tasks and influence outcomes. It is an integral element in the adaptation and coping strategies of individuals with chronic health conditions.^24^ Self-efficacy was chosen among other patient-reported psychological construct variables, such as self-esteem or self-confidence,^25^^,^^26^ on the basis of previously published literature in adolescents with chronic heart failure^27^ and in an ACHD patient cohort.^17^ The General Self-Efficacy Questionnaire (GSE) has proven to be a valuable tool to assess self-efficacy. The GSE comprises 10 items that gauge an individual’s general sense of self-efficacy in managing life’s challenges.^28^ A higher score indicates a higher level of self-efficacy. All items of all patient questionnaires were filled out completely, but 0, 8, and 17 patients failed to fill out the questionnaires at baseline, 1-year, and 3-year follow-up, respectively.

### Statistical methods

Descriptive statistics were reported as mean values and standard deviation. All endpoints were analyzed by intention-to-treat, using 2-sided superiority testing with α set at 5%, so that *P* values of <0.050 were considered significant.

The calculation of the sample size for the SERVE trial was based on the primary endpoint of a change in RV end-systolic volume from baseline to follow-up. The performed calculation led to an estimated requirement of 98 patients overall with 49 patients for each group.^12^ For the aim of this *post hoc* analysis, the appropriateness of the sample number was checked. To distinguish an LAS score difference of at least 5 points between baseline and follow-up, a sample size of 73 patients was calculated, setting the β error at 80%.

The SERVE trial cohort of 100 patients was herewith sufficient also taking into account the dropouts at follow-up.

For continuous variables, differences between mean values were analyzed using unpaired Student *t* tests when comparing 2 groups. Comparisons between 3 or more groups were performed using unifactorial analysis of variance (ANOVA) as a first step. Longitudinal comparisons between group pairs were performed using a paired Student *t* test. Inferential statistics were performed using the χ^2^ test for nominal or ordinal level data.

Correlations analyses were performed using the Pearson correlation test for continuous variables and the Spearman correlation test for nominal or ordinal level data. The strength of correlation was expressed with the *r* value for the Pearson test and with the ρ value for the Spearman test. Unifactorial and multifactorial linear regression models were used to identify predictors. The amount of variability explained by the predictor was expressed using the *r*^2^ value.

A comparative analysis was performed to contrast patients whose QoL on the LAS scale improved over the 3-year follow-up with patients whose QoL on the LAS scale remained stable or diminished. The improvement was defined as clinically significant if the LAS score increased by at least 10 points at follow-up. Similarly, a clinically significant worsening was defined as a reduction of at least 10 points in the LAS score at 3-year follow-up.

Data analysis was performed using IBM SPSS Statistics for Windows, version 25 (IBM Corp, Armonk, NY).

## Results

### Cohort characteristics

**Figure 1:**
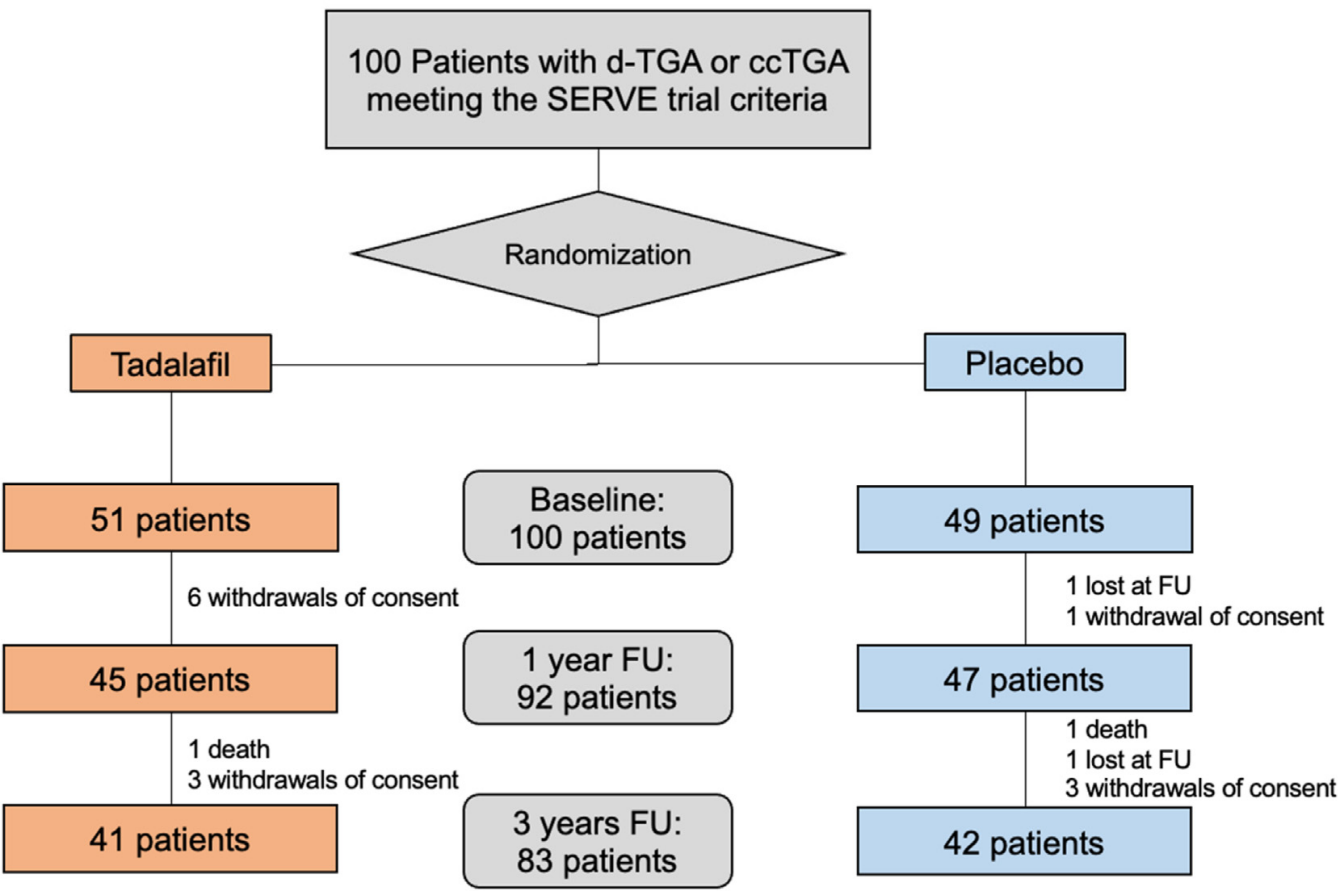
Flowchart of participants in the SERVE study. ccTGA, congenitally corrected transposition of the great arteries; d-TGA, dextro–transposition of the great arteries after atrial switch operation; FU, follow-up.

Of the 100 patients included in the SERVE trial, 51 patients were assigned to the treatment group with tadalafil, whereas 49 patients were allocated to the placebo group (Fig. 1). Enrollment occurred between October 18, 2017, and August 6, 2018.^11^ A total of 75% of the patients were affected by d-TGA and had undergone an atrial switch procedure. The remaining 25% were affected by ccTGA. The average age at baseline of study participants was 40.7 ± 10.7 years. Common comorbidities in this cohort were tachyarrhythmias (34%), ventricular septal defects (21%), baffle leaks (30%), and significant tricuspid valve regurgitation (34%), as summarized in Table 1.

**Table 1:** Main clinical characteristics and the QoL parameters at baseline in the overall population of the SERVE trial as well as for each of the treatment groups

Baseline characteristics			
	Overall	Tadalafil	Placebo
N	100	51	49
Clinical characteristics			
Female, n (%)	33 (33)	14 (27)	19 (39)
Age (y)	40.7 ± 10.7	41.3 ± 10.2	40.1 ± 11.4
Atrial switch procedure, n (%)	75 (75)	38 (75)	37 (76)
Congenitally corrected TGA, n (%)	25 (25)	13 (25)	12 (24)
NYHA, n (%)			
I	83 (83)	43 (84)	40 (82)
II	13 (13)	6 (12)	7 (14)
III	4 (4)	2 (4)	2 (4)
IV	0	0	0
History of tachyarrhythmias, n (%)	34 (34)	19 (37)	15 (31)
Previous pacemaker or AICD implantation, n (%)	21 (21)	11 (22)	10 (20)
Moderate or severe tricuspid regurgitation, n (%)	34 (34)	15 (29)	19 (39)
Ventricular septal defect, n (%)	21 (21)	12 (24)	9 (18)
Residual atrial shunt, n (%)	30 (30)	12 (24)	18 (37)
Palliation procedure before Mustard/Senning repair, n (%)	59 (59)	28 (55)	31 (63)
Prior stroke, n (%)	14 (14)	5 (10)	9 (18)
Patient-reported outcomes			
LAS (0-100)	79.1 ± 13.6	75.5 ± 14.8	83.0 ± 10.7
SWLS (5-35)	27.9 ± 5.3	26.6 ± 6.0	29.2 ± 4.0
GSE (10-40)	31.9 ± 4.3	31.1 ± 4.6	32.3 ± 3.9

Age, GSE score, LAS score, and SWLS score are expressed as mean value ± standard deviation. The frequency of each characteristic expressed in percentage of the whole group for the overall cohort, the tadalafil group, and the placebo group, respectively, is enclosed within parentheses.

AICD, automatic implantable cardioverter defibrillator; GSE, General Self-Efficacy Questionnaire; LAS, Linear Analog Scale; NYHA, New York Heart Association; QoL, quality of life; SWLS, Satisfaction with Life Scale; TGA, transposition of the great arteries.

The follow-up was completed successfully in 92 patients after the first year and in 83 patients after 3 years. The reasons for patient dropout were withdrawal of consent (13 patients), loss at follow-up (2 patients), and death (2 patients).

### QoL levels at baseline

At baseline, the LAS score was similarly distributed over both sexes (*P* = 0.619, see Table 1) and over the different age groups both in ccTGA and in d-TGA patients (Table 2). Patients with a lower NYHA class (1 and 2) reported a better QoL on the LAS score than patients with an NYHA class 3 (Table 2, ANOVA *P* value <0.001), whereas no significant difference could be seen between LAS scores in NYHA 1 and NYHA 2 patients. None of the recruited patients were in functional NYHA class 4 either at baseline or at follow-up.

**Table 2:** QoL levels on the LAS in different clusters at baseline according to original anatomy, age group, sex, and NYHA class

LAS score	Age groups (y)				Sex	
Anatomy	18-35	36-49		Over 50	Male	Female

ccTGA	73.2 ± 9.3 (n = 5)	83.6 ± 5.5 (n = 7)		73.6 ± 19.5 (n = 13)	79.3 ± 10.4 (n = 16)	71.1 ± 21.2 (n = 9)
d-TGA	82.3 ± 11.9 (n = 29)	78.3 ± 14.4 (n = 39)		80.7 ± 6.1 (n = 7)	79.4 ± 12.7 (n = 51)	81.5 ± 13.4 (n = 24)
All anatomies	80.9 ± 11.9 (n = 34)	79.1 ± 13.6 (n = 46)		76.1 ± 16.2 (n = 20)	79.4 ± 12.2 (n = 67)	78.6 ± 15.9 (n = 33)

LAS score	NYHA class			3-year follow-up		

Anatomy	NYHA 1	NYHA 2	NYHA 3	Baseline	1 year	3 years

ccTGA	79.4 ± 12.0 (n = 19)	78.8 ± 8.5 (n = 4)	42.5 ± 17.7 (n = 2)	76.3 ± 15.3 (n = 25)	75.9 ± 12.6 (n = 20)	77.1 ± 14.4 (n = 16)
d-TGA	81.0 ± 12.8 (n = 64)	78.9 ± 9.6 (n = 9)	56.0 ± 8.5 (n = 2)	80.1 ± 12.9 (n = 75)	81.1 ± 12.9 (n = 72)	79.7 ± 13.4 (n = 67)
All anatomies	80.6 ± 12.6 (n = 83)	78.8 ± 8.9 (n = 13)	49.3 ± 13.7 (n = 4)	79.1 ± 13.6 (n = 100)	79.9 ±12.9 (n = 92)	79.2 ± 13.6 (n = 83)

The values are expressed as mean value ± standard deviation. On the right side of the table, the LAS development over the follow-up controls is shown for the whole population and for the ccTGA and d-TGA subgroups.

ccTGA, congenitally corrected transposition of the great arteries; d-TGA, dextro–transposition of the great arteries; LAS, Linear Analog Scale; NYHA, New York Heart Association; QoL, quality of life.

### QoL levels during follow-up

**Figure 2:**
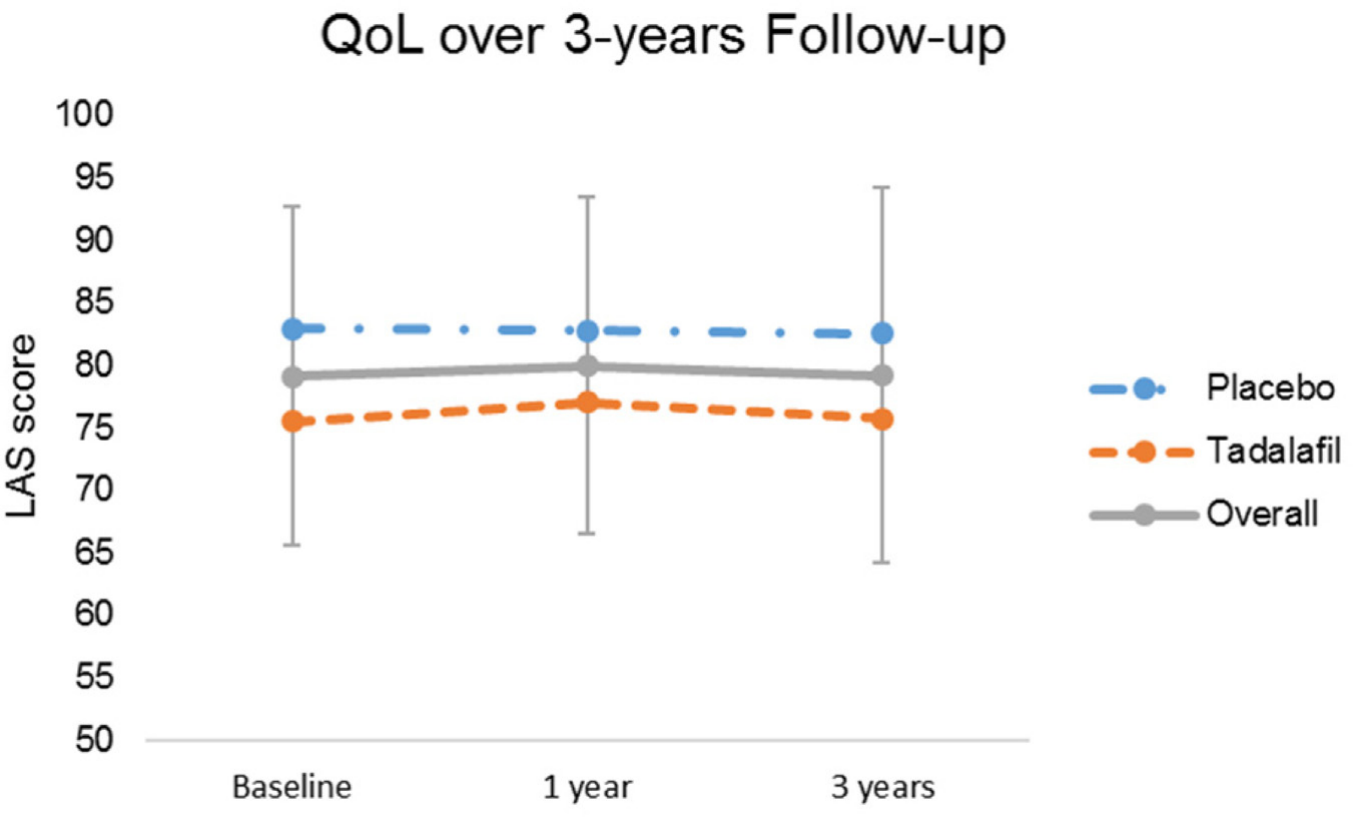
LAS scores and their changes over the 3-year follow-up in the intervention and placebo groups. The points indicate mean values, and the bars represent the standard deviation. Sample sizes for baseline, 1 year, and 3 years are n = 100, 92, and 83 for the overall cohort, n = 51, 45, and 41 for tadalafil, and n = 49, 47, and 42 for placebo, respectively. LAS, Linear Analog Scale; QoL, quality of life.

A total of 29 patients experienced a decrease in LAS score over the 3-year period and 37 patients experienced an increase. Overall, the mean LAS score was not significantly different both after 1 year and after 3 years (ANOVA *P* value 0.900; Fig. 2). The follow-up also did not result in significant changes in the mean SWLS score, which amounted to 28.4 ± 4.7 after 1 year and 28.1 ± 4.6 after 3 years (ANOVA *P* value 0.720).

**Figure 3:**
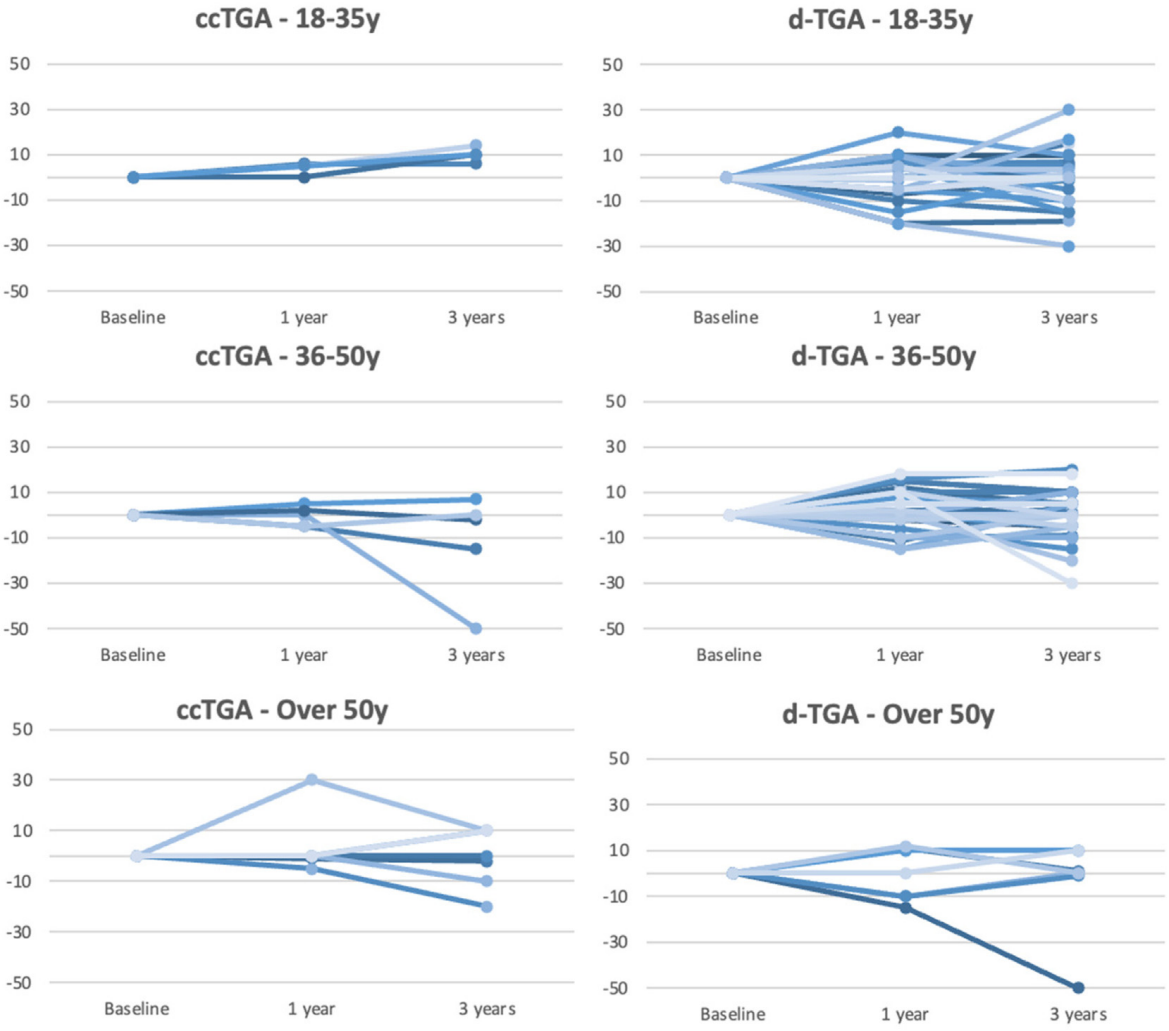
Variation of the LAS scores over the 3-year follow-up in subgroups according to the original anatomy and to the age category. Each line represents a single patient. ccTGA, congenitally corrected transposition of the great arteries; d-TGA, dextro–transposition of the great arteries; LAS, Linear Analog Scale.

The LAS score did not change significantly over the observation period both in patients with ccTGA and in patients with d-TGA and atrial switch (Table 2). When clustered in different age groups, a comparable pattern over the 3-year follow-up was observed (Fig. 3).

### Impact of self-efficacy on QoL

**Figure 4:**
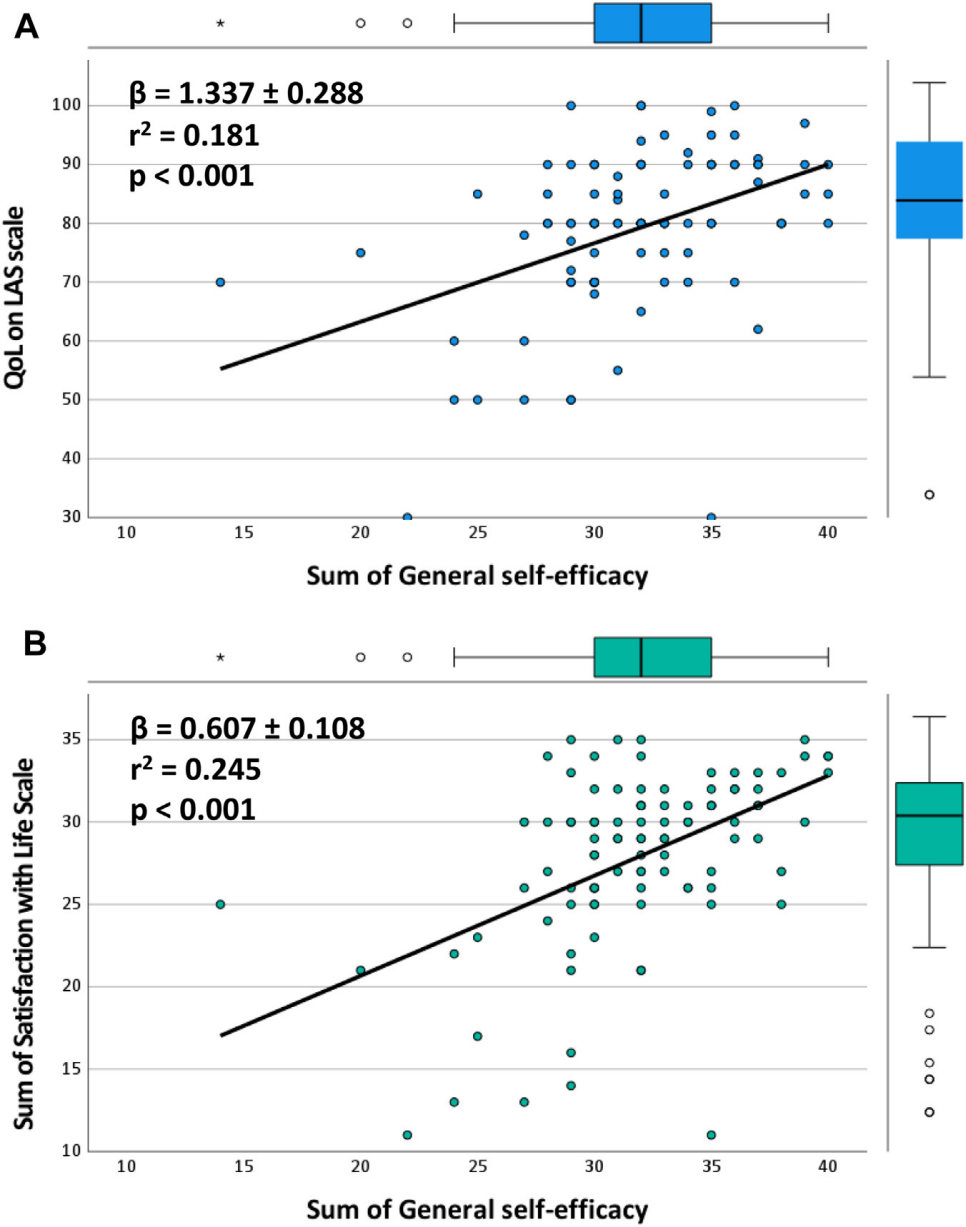
Regression analysis performed to investigate the role of GSE as a predictor of QoL. (**A**) Shows the regression line for GSE and LAS score; (**B**) shows the regression line for GSE and SWLS score. A linear correlation is shown according to which higher GSE values predict a higher QoL level. The β coefficient indicates the slope of the curve, the *r* value indicates the strength of the linear relationship between the 2 studied variables, and the *r*^2^ value indicates the proportion of the QoL variation that is predictable from the GSE. The points indicate the values recorded in the single patients. The sample size is n = 100 for each of panels (**A**) and (**B**). GSE, General Self-Efficacy Questionnaire; LAS, Linear Analog Scale; QoL, quality of life; SWLS, Satisfaction with Life Scale.

The baseline mean GSE value was 31.9 ± 4.3. The GSE showed a correlation with the QoL values, where a higher GSE score correlated with higher QoL levels. A linear regression analysis showed a progressive increase of QoL values on both LAS (*r*^2^ = 0.181, β coefficient = +1.337 ± 0.288, *P* < 0.001) and SWLS (*r*^2^ = 0.245, β coefficient = +0.607 ± 0.108, *P* < 0.001) scales with increasing GSE scores (Fig. 4).

### Clinical predictors of QoL

Predefined clinical parameters were tested for correlation with the QoL levels on LAS scales. No correlation emerged between QoL and age, gender, significant tricuspid valve regurgitation, RV ejection fraction, or positive history of tachyarrhythmias (Table 3).

**Table 3:** Calculated correlation, univariable regression, and multivariable regression analysis results between QoL and clinical, imaging, CPET, and blood test parameters, at baseline (N = 100)

Examined parameters	QoL on LAS scale (correlation analysis)		QoL on LAS scale (univariable regression analysis)			QoL on LAS scale (multivariable regression analysis)		
	Correlation coefficient	*P* value	*r*^2^	β	*P* value	*r*^2^	β	*P* value
Clinical								
General self-efficacy	0.444	<0.001	0.181	1.337 ± 0.288	<0.001	0.283	1.231 ± 0.272	<0.001
NYHA class	−0.250	0.012	0.132	−9.880 ± 2.563	<0.001		−8.765 ± 2.354	<0.001
Age	−0.160	0.113	–	–	–	–	–	–
Sex	−0.116	0.250	–	–	–	–	–	–
Atrial switch procedure	0.106	0.294	–	–	–	–	–	–
Previous tachyarrhythmias	−0.140	0.165	–	–	–	–	–	–
Imaging								
MRI RVEDV	−0.208	0.038	0.015	−0.026 ± 0.021	0.217	–	–	–
MRI RV ESV	−0.140	0.165	–	–	–	–	–	–
MRI RV EF	0.011	0.913	–	–	–	–	–	–
CPET								
Watt at peak	0.243	0.015	0.072	0.068 ± 0.025	0.007	–	–	–
Peak VO2	0.245	0.015	0.062	0.454 ± 0.180	0.013	–	–	–
VE/CO2 slope	−0.150	0.137	–	–	–	–	–	–
Blood test								
NT-proBNP	−0.224	0.025	0.058	−0.004 ± 0.002	0.016	–	–	–
Intercept	–	–	–	–	–	–	41.757 ± 8.808	–

The correlation coefficients (*r* or ρ) and their respective significance *P* values are obtained through the Pearson correlation test for continuous variables and through the Spearman correlation test for ordinal or nominal variables. Linear regression models were applied for both univariate and multivariable regression analysis: the resulting *r*^2^ values and β coefficients are reported. Statistical significance was set for *P* values <0.050.

CPET, cardiopulmonary exercise testing; EF, ejection fraction; ESV, end-systolic volume; MRI, magnetic resonance imaging; NT-proBNP, N-terminal pro–B-type natriuretic peptide; NYHA, New York Heart Association; QoL, quality of life; RV, right ventricle; RVEDV, right ventricular end-diastolic volume; VE/CO2, minute ventilation to carbon dioxide production slope; VO2, oxygen uptake.

Data from functional tests showed that the NYHA class and the cardiorespiratory fitness correlated with QoL. Lower NYHA class (ρ = −0.363, *P* = 0.012), higher maximum work rate (*r* = 0.308, *P* = 0.002), and higher maximum oxygen uptake (*r* = 0.245, *P* = 0.015) correlated with higher LAS scores.

End-diastolic RV volumes measured by CMR showed significant correlation with QoL, where smaller volumes were associated with better QoL. Also, patients with a higher than median QoL on the LAS score had significantly lower N-terminal pro–B-type natriuretic peptide (NT-proBNP) concentrations than the rest of the cohort. Further details on the correlation analyses are provided in Table 3.

In a second step, a single regression analysis was performed to investigate a possible predictor role of the variables showing significant correlation with QoL on the LAS scale. Here, higher NYHA class, lower NT-proBNP levels, higher maximum work rate, and higher maximum oxygen uptake demonstrated a significant association with higher QoL levels (Table 3), but not end-diastolic RV volumes measured by CMR (*r*^2^ = 0.015, β coefficient = −0.026 ± 0.021, *P* = 0.217).

In a multivariable logistic regression analysis, only the GSE value and NYHA class were independent predictors of QoL. The model including GSE and NYHA as predictors explained only 28.3% of the QoL variability (Table 3).

### Predictors of QoL improvement or worsening

No significant differences were observed among these 3 subgroups considering age, sex, original anatomy, NYHA class, GSE score, peak oxygen uptake, RV end-diastolic volume, NT-proBNP levels, or intervention group (Table 4). Similarly, no significant differences between the groups could be observed as for the history of arrhythmias, history of previous palliative surgery, presence of a residual intracardiac shunt, or history of stroke.

**Table 4:** Baseline clinical characteristics of patients whose QoL on LAS scale declined, remained stable, or increased over the 3-year follow-up period (N = 83)

Characteristics	QoL decrease (reduction ≥10 points)	Stable QoL (variation <10 points)	QoL increase (increase ≥10 points)	*P* value
Overall, n (%)	18 (21.7)	44 (53.0)	21 (25.3)	–
Randomization group, n (%)				
Tadalafil	9 (10.8)	21 (25.3)	11 (13.2)	0.939
Placebo	9 (10.8)	23 (27.7)	10 (12.0)	
Original anatomy, n (%)				
ccTGA	4 (4.8)	6 (7.2)	6 (7.2)	0.339
d-TGA	14 (16.9)	38 (45.8)	15 (18.1)	
Sex, n (%)				
Female	8 (9.6)	12 (14.5)	7 (8.4)	0.422
Male	10 (12.0)	32 (38.6)	14 (16.9)	
Baseline NYHA class, n (%)				
I	14 (16.9)	40 (48.2)	15 (18.1)	0.115
II or higher	4 (4.8)	4 (4.8)	6 (7.2)	
3-year FU NHYA class, n (%)				
I	14 (16.9)	37 (44.6)	17 (20.5)	0.834
II or higher	4 (4.8)	7 (8.4)	4 (4.8)	
Age (y), mean ± standard deviation	38.8 ± 9.9	38.7 ± 8.8	41.1 ± 12.9	0.658
Baseline peak VO2 (mL/min/kg), mean ± standard deviation	22.2 ± 7.0	26.2 ± 7.5	23.5 ± 7.6	0.112
Peak VO2 variation (mL/min/kg), mean ± standard deviation	1.1 ± 4.1	−2.0 ± 4.1	−1.1 ± 4.2	0.138
Baseline RVEDV (mL), mean ± standard deviation	215.9 ± 48.9	249.7 ± 70.0	230.2 ± 54.3	0.132
RVEDV variation (mL), mean ± standard deviation	4.8 ± 26.5	3.7 ± 25.6	−0.6 ± 24.6	0.762
Baseline NT-proBNP (pg/mL), mean ± standard deviation	256.8 ± 288.5	499.2 ± 892.8	447.1 ± 365.2	0.711
NT-proBNP variation (pg/mL), mean ± standard deviation	67.7 ± 175.6	−72.6 ± 566.2	89.7 ± 459.0	0.392
Baseline GSE (n/40), mean ± standard deviation	32.7 ± 4.1	32.1 ± 3.2	31.6 ± 6.2	0.461
GSE variation (n/40), mean ± standard deviation	−0.7 ± 3.4	−0.5 ± 4.4	0.3 ± 3.1	0.637

No statistically significant differences were observed among the 3 groups for the analyzed variables. A variation was defined as statistically significant for *P* values <0.050.

ccTGA, congenitally corrected transposition of the great arteries; d-TGA, dextro–transposition of the great arteries; FU, follow-up; GSE, general self-efficacy; LAS, Linear Analog Scale; NT-proBNP, N-terminal pro–B-type natriuretic peptide; NYHA, New York Heart Association; QoL, quality of life; RVEDV, right ventricular end-diastolic volume; SWLS, Satisfaction with Life Scale; VO2, oxygen uptake.

## Discussion

The SERVE trial provided standardized clinical, anatomic, and functional data on a prospective cohort of patients with systemic RV over a 3-year observational period. The SERVE trial failed to demonstrate a significant effect of tadalafil on QoL in the studied cohort, in contrast to the demonstrated beneficial effect of tadalafil on QoL in other conditions such as pulmonary hypertension or hepatic cirrhosis.^29^^,^^30^ The present study demonstrates that these ACHD patients reported a high and stable QoL during the study period. The mean scores on both LAS and SWLS were in line with the results of the APPROACH-IS study comparing QoL in adults with congenital heart diseases in 15 countries.^8^ In APPROACH-IS, the median LAS score was 80.0 and the median SWLS score was 27.0 in a population with a median age of 32 years. Although the majority of patients included in the APPROACH-IS study had noncomplex cardiac defects, the results in our study population with complex congenital heart disease are very similar (mean LAS: 79.1 and mean SLWS: 27.9). This is pleasingly surprising, considering the impaired long-term outcome of these patients. Not only was baseline QoL comparable to the APPROACH-IS study, but its predictors were also identical. Similar to the APPROACH-IS cohort, the majority of the systemic RV patients recruited in the SERVE trial had an NYHA class 1 or 2 (96% in the SERVE vs 89% in the APPROACH-IS study).^7^^,^^8^ The NYHA functional class did correlate with the QoL level in both studies, whereas sex or type of defect did not.^8^ The present findings are also comparable with historical data from Moons et al.,^31^ investigating the QoL after the Mustard or Senning procedure for d-TGA 20 years ago where a median LAS score of 80 was reported for the 89 patient enrolled in the study. Of note, in this study, patients were much younger (median age of 24 years) than our patient cohort (mean age 41 years), indicating that QoL seems not necessarily to deteriorate with age, as also expressed by the overall QoL stability during the SERVE observation period of 3 years.

In contrast to the previously mentioned studies, the SERVE trial offered the unique opportunity to study QoL prospectively, over a period of 3 years. It was very reassuring to document that the overall QoL remained stable during this time. It should be remarked, though, that the study cohort was selected using exclusion criteria that excluded the most severe end of the clinical spectrum, because the patients with severe liver or renal dysfunction were excluded, as well as patients with expected heart transplant within 6 months from baseline.

Patients with a systemic RV are at risk for heart failure or clinically significant arrhythmias over the years, and the majority of these patients have experienced at least one of such complications by the fifth decade of life.^1^^,^^32^^,^^33^ In line to these data, 34% of the SERVE patients suffered at least an adverse event during the 3 years of follow-up.^11^ Despite this, age does not correlate with QoL, as our data show, and the comparison of the QoL of SERVE patients with the younger populations from the APPROACH-IS and from the aforementioned Belgian cohort of d-TGA patients serves to reinforce these findings.^8^^,^^31^

The well-established but subjective NYHA classification for dyspnea appeared to be a consistent and significant QoL predictor also in ACHD patients, despite that this classification was originally developed for patients with acquired heart disease. Of note, none of the advanced imaging parameters of right ventricular function were independently associated with QoL. This might suggest that the assessment of NYHA class includes factors affecting QoL beyond the focused quantification of RV function. Not surprisingly, a significant correlation could be highlighted between the functional capacity in cardiopulmonary exercise testing and QoL. These findings suggest that the main factor influencing patients’ QoL may be mainly their functional capacity and not a single marker of systemic ventricular function.

Another important finding of our study was the identification of the global self-efficacy as an independent predictor of QoL. A growing body of research suggests a correlation between an individual’s self-efficacy and QoL in different patient populations.^34^, ^35^, ^36^ Higher levels of self-efficacy were described to be associated with improved psychological well-being, enhanced adherence to medical recommendations, and a more optimistic outlook on life.^37^ Recently, the GSE score was found to be also a predictor of QoL in ACHD patients.^17^ Thomet et al.^17^ describe a mean GSE score of 30.1 ± 3.3 in a cohort of 454 ACHD patients recruited in 2 centers in Canada and Switzerland, with a score of 29.5 ± 5.7 in the subgroup affected by complex congenital heart defects. These numbers correspond well to our cohort of complex ACHD patients and a GSE score of 31.9 ± 4.3 at baseline and 31.8 ± 4.6 after 3 years. Consequently, as outlined by Thomet et al.,^17^ an improvement in self-efficacy may result in an improvement in QoL and therefore offers another approach to improve the outcome in these patients, beyond purely medical means.

Regarding the factors related to QoL variation over time, our study did not find any significant difference between patients with a QoL improvement and patients with a QoL worsening, as far as clinical history, imaging or functional parameters, or medical therapy is concerned. It has to be stated, though, that the study was underpowered for that scope both as for sample size dimension and as for follow-up duration.

Finally, it is interesting to observe that the population described here did not show significant changes in the QoL scores at 1-year and 3-year follow-ups, even though the COVID-19 pandemic occurred in between. A possible explanation may be found in the fact that the cohort included individuals who were already chronically medicalized and had an established trust relationship with their respective ACHD health care teams.

### Limitations

The presented results are subject to limitations related to the study design. First, the SERVE trial was designed to establish the effects of tadalafil on the systemic right ventricular function and dimension compared with placebo. The QoL levels were not designed to be a primary outcome; therefore, sample size was not chosen to detect changes in QoL. This, also in the light of a recorded 17% patient loss at follow-up, may limit the interpretation and the generalizability of the results. Also, the study design carries intrinsic limitations related to a *post hoc* analysis such as the increased type II error and the retrospective analysis of the data, with the hypothesis being specified only after the data collection.

Secondly, social factors were not documented. While being a job seeker and never having married were suggested to be a predictor of poor QoL in other studies, these variables were not assessed in the SERVE trial.^8^ Further, the study did not consider the possible role of psychological factors as perceived illness, spirituality, and religion. Lastly, we did not consider country-specific differences, which might exist between patients living in Switzerland and in Austria.

## Conclusions

Adult patients with ccTGA or d-TGA and a systemic RV report a high level of QoL with a stable course over a 3-year follow-up. Global self-efficacy and NYHA class are the main predictors of QoL in this cohort of patients. No clinical characteristics could be detected that might predict an improvement or a worsening of the QoL level over the 3-year follow-up.
