# Supplementary material for: Stable Longitudinal Quality of Life in the SERVE Trial Among Adults With Transposition of the Great Arteries and a Systemic Right Ventricle
Source: CJC Pediatr Congenit Heart Dis. 2024 Dec 12;4(2):81–91. doi: 10.1016/j.cjcpc.2024.12.001 (PMC12131024; doi:10.1016/j.cjcpc.2024.12.001)
Supplement: Supplementary Data [file mmc1.docx]

# Reporting checklist for randomised trial.

Based on the CONSORT guidelines.

## Instructions to authors

Complete this checklist by entering the page numbers from your manuscript where readers will find each of the items listed below.

Your article may not currently address all the items on the checklist. Please modify your text to include the missing information. If you are certain that an item does not apply, please write "n/a" and provide a short explanation.

Upload your completed checklist as an extra file when you submit to a journal.

In your methods section, say that you used the CONSORTreporting guidelines, and cite them as:

Schulz KF, Altman DG, Moher D, for the CONSORT Group. CONSORT 2010 Statement: updated guidelines for reporting parallel group randomised trials

|  |  | Reporting Item | Page Number |
| --- | --- | --- | --- |
| **Title and Abstract** |  |  |  |
| Title | [#1a](https://www.goodreports.org/reporting-checklists/consort/info/#1a) | Identification as a randomized trial in the title. | 1 |
| Abstract | [#1b](https://www.goodreports.org/reporting-checklists/consort/info/#1b) | Structured summary of trial design, methods, results, and conclusions | 4 |
| **Introduction** |  |  |  |
| Background and objectives | [#2a](https://www.goodreports.org/reporting-checklists/consort/info/#2a) | Scientific background and explanation of rationale | 7 (see references paper on methods and main paper with results, both cited) |
| Background and objectives | [#2b](https://www.goodreports.org/reporting-checklists/consort/info/#2b) | Specific objectives or hypothesis | 7-9 (see references paper on methods and main paper with results, both cited) |
| **Methods** |  |  |  |
| Trial design | [#3a](https://www.goodreports.org/reporting-checklists/consort/info/#3a) | Description of trial design (such as parallel, factorial) including allocation ratio. | 7-9 (see references paper on methods and main paper with results, both cited) |
| Trial design | [#3b](https://www.goodreports.org/reporting-checklists/consort/info/#3b) | Important changes to methods after trial commencement (such as eligibility criteria), with reasons | n/a |
| Participants | [#4a](https://www.goodreports.org/reporting-checklists/consort/info/#4a) | Eligibility criteria for participants | 8 (see references paper on methods and main paper with results, both cited) |
| Participants | [#4b](https://www.goodreports.org/reporting-checklists/consort/info/#4b) | Settings and locations where the data were collected | 7-9 (see references paper on methods and main paper with results, both cited) |
| Interventions | [#5](https://www.goodreports.org/reporting-checklists/consort/info/#5) | The experimental and control interventions for each group with sufficient details to allow replication, including how and when they were actually administered | 7-9 and 12 (see references paper on methods and main paper with results, both cited) |
| Outcomes | [#6a](https://www.goodreports.org/reporting-checklists/consort/info/#6a) | Completely defined prespecified primary and secondary outcome measures, including how and when they were assessed | 7-12 (see references paper on methods and main paper with results, both cited) |
| Sample size | [#7a](https://www.goodreports.org/reporting-checklists/consort/info/#7a) | How sample size was determined. | 10-11 (see references paper on methods and main paper with results, both cited) |
| Sample size | [#7b](https://www.goodreports.org/reporting-checklists/consort/info/#7b) | When applicable, explanation of any interim analyses and stopping guidelines | n/a |
| Randomization - Sequence generation | [#8a](https://www.goodreports.org/reporting-checklists/consort/info/#8a) | Method used to generate the random allocation sequence. | 7-9 (see references paper on methods and main paper with results, both cited) |
| Randomization - Sequence generation | [#8b](https://www.goodreports.org/reporting-checklists/consort/info/#8b) | Type of randomization; details of any restriction (such as blocking and block size) | 7-9 (see references paper on methods and main paper with results, both cited) |
| Randomization - Allocation concealment mechanism | [#9](https://www.goodreports.org/reporting-checklists/consort/info/#9) | Mechanism used to implement the random allocation sequence (such as sequentially numbered containers), describing any steps taken to conceal the sequence until interventions were assigned | 7-9 (see references paper on methods and main paper with results, both cited) |
| Randomization - Implementation | [#10](https://www.goodreports.org/reporting-checklists/consort/info/#10) | Who generated the allocation sequence, who enrolled participants, and who assigned participants to interventions | 7-9 (see references paper on methods and main paper with results, both cited) |
| Blinding | [#11a](https://www.goodreports.org/reporting-checklists/consort/info/#11a) | If done, who was blinded after assignment to interventions (for example, participants, care providers, those assessing outcomes) and how. | 7-9 (see references paper on methods and main paper with results, both cited) |
| Blinding | [#11b](https://www.goodreports.org/reporting-checklists/consort/info/#11b) | If relevant, description of the similarity of interventions | 7-9 (see references paper on methods and main paper with results, both cited) |
| Statistical methods | [#12a](https://www.goodreports.org/reporting-checklists/consort/info/#12a) | Statistical methods used to compare groups for primary and secondary outcomes | 9-11 (see references paper on methods and main paper with results, both cited) |
| Statistical methods | [#12b](https://www.goodreports.org/reporting-checklists/consort/info/#12b) | Methods for additional analyses, such as subgroup analyses and adjusted analyses | 9-11 (see references paper on methods and main paper with results, both cited) |
| Outcomes | [#6b](https://www.goodreports.org/reporting-checklists/consort/info/#6b) | Any changes to trial outcomes after the trial commenced, with reasons | 7-9 (see references paper on methods and main paper with results, both cited) |
| **Results** |  |  |  |
| Participant flow diagram (strongly recommended) | [#13a](https://www.goodreports.org/reporting-checklists/consort/info/#13a) | For each group, the numbers of participants who were randomly assigned, received intended treatment, and were analysed for the primary outcome | 12 (see references paper on methods and main paper with results, both cited) |
| Participant flow | [#13b](https://www.goodreports.org/reporting-checklists/consort/info/#13b) | For each group, losses and exclusions after randomization, together with reason | 12 (see references paper on methods and main paper with results, both cited) |
| Recruitment | [#14a](https://www.goodreports.org/reporting-checklists/consort/info/#14a) | Dates defining the periods of recruitment and follow-up | 12 (see references paper on methods and main paper with results, both cited) |
| Recruitment | [#14b](https://www.goodreports.org/reporting-checklists/consort/info/#14b) | Why the trial ended or was stopped | n/a |
| Baseline data | [#15](https://www.goodreports.org/reporting-checklists/consort/info/#15) | A table showing baseline demographic and clinical characteristics for each group | 12-13 (see references paper on methods and main paper with results, both cited) |
| Numbers analysed | [#16](https://www.goodreports.org/reporting-checklists/consort/info/#16) | For each group, number of participants (denominator) included in each analysis and whether the analysis was by original assigned groups | 12-13 (see references paper on methods and main paper with results, both cited) |
| Outcomes and estimation | [#17a](https://www.goodreports.org/reporting-checklists/consort/info/#17a) | For each primary and secondary outcome, results for each group, and the estimated effect size and its precision (such as 95% confidence interval) | n/a |
| Outcomes and estimation | [#17b](https://www.goodreports.org/reporting-checklists/consort/info/#17b) | For binary outcomes, presentation of both absolute and relative effect sizes is recommended | n/a |
| Ancillary analyses | [#18](https://www.goodreports.org/reporting-checklists/consort/info/#18) | Results of any other analyses performed, including subgroup analyses and adjusted analyses, distinguishing pre-specified from exploratory | n/a |
| Harms | [#19](https://www.goodreports.org/reporting-checklists/consort/info/#19) | All important harms or unintended effects in each group (For specific guidance see CONSORT for harms) | n/a |
| **Discussion** |  |  |  |
| Limitations | [#20](https://www.goodreports.org/reporting-checklists/consort/info/#20) | Trial limitations, addressing sources of potential bias, imprecision, and, if relevant, multiplicity of analyses | 19-20 |
| Interpretation | [#22](https://www.goodreports.org/reporting-checklists/consort/info/#22) | Interpretation consistent with results, balancing benefits and harms, and considering other relevant evidence | 16-19 |
| Registration | [#23](https://www.goodreports.org/reporting-checklists/consort/info/#23) | Registration number and name of trial registry | 8 |
| Generalisability | [#21](https://www.goodreports.org/reporting-checklists/consort/info/#21) | Generalisability (external validity, applicability) of the trial findings | 16-19 |
| **Other information** |  |  |  |
| Interpretation | [#22](https://www.goodreports.org/reporting-checklists/consort/info/#22) | Interpretation consistent with results, balancing benefits and harms, and considering other relevant evidence | 16-19 |
| Registration | [#23](https://www.goodreports.org/reporting-checklists/consort/info/#23) | Registration number and name of trial registry | 8 |
| Protocol | [#24](https://www.goodreports.org/reporting-checklists/consort/info/#24) | Where the full trial protocol can be accessed, if available | 7-9 (see references paper on methods and main paper with results, both cited) |
| Funding | [#25](https://www.goodreports.org/reporting-checklists/consort/info/#25) | Sources of funding and other support (such as supply of drugs), role of funders | 20 |

Notes:

- 2a: 6-7 (see references paper on methods and main paper with results, both cited)
- 2b: 7-8 (see references paper on methods and main paper with results, both cited)
- 3a: 7-8 (see references paper on methods and main paper with results, both cited)
- 4a: 7-8 (see references paper on methods and main paper with results, both cited)
- 4b: 7-8 (see references paper on methods and main paper with results, both cited)
- 5: 7-8 (see references paper on methods and main paper with results, both cited)
- 6a: 7-8 (see references paper on methods and main paper with results, both cited)
- 7a: 7-8 (see references paper on methods and main paper with results, both cited)
- 8a: 7-8 (see references paper on methods and main paper with results, both cited)
- 8b: 7-8 (see references paper on methods and main paper with results, both cited)
- 9: 7-8 (see references paper on methods and main paper with results, both cited)
- 10: 7-8 (see references paper on methods and main paper with results, both cited)
- 11a: 7-8 (see references paper on methods and main paper with results, both cited)
- 11b: 7-8 (see references paper on methods and main paper with results, both cited)
- 12a: 7-8 (see references paper on methods and main paper with results, both cited)
- 12b: 7-8 (see references paper on methods and main paper with results, both cited)
- 13a: 7-11 (see references paper on methods and main paper with results, both cited)
- 13b: 7-11 (see references paper on methods and main paper with results, both cited)
- 14a: 7-11 (see references paper on methods and main paper with results, both cited)
- 15: 7-11 (see references paper on methods and main paper with results, both cited)
- 16: 7-11 (see references paper on methods and main paper with results, both cited)
- 24: 7-8 (see references paper on methods and main paper with results, both cited)
- 6b: 7-8 (see references paper on methods and main paper with results, both cited) The CONSORT checklist is distributed under the terms of the Creative Commons Attribution License CC-BY. This checklist was completed on 17. May 2024 using <https://www.goodreports.org/>, a tool made by the [EQUATOR Network](https://www.equator-network.org) in collaboration with [Penelope.ai](https://www.penelope.ai)
